# Identification of Gene Expression Differences between Lymphangiogenic and Non-Lymphangiogenic Non-Small Cell Lung Cancer Cell Lines

**DOI:** 10.1371/journal.pone.0150963

**Published:** 2016-03-07

**Authors:** Erin Regan, Robert C. Sibley, Bercin Kutluk Cenik, Asitha Silva, Luc Girard, John D. Minna, Michael T. Dellinger

**Affiliations:** 1 Hamon Center for Therapeutic Oncology Research, UT Southwestern Medical Center, Dallas, Texas, United States of America; 2 Department of Pharmacology, UT Southwestern Medical Center, Dallas, Texas, United States of America; 3 Department of Internal Medicine, UT Southwestern Medical Center, Dallas, Texas, United States of America; 4 Division of Surgical Oncology, Department of Surgery, UT Southwestern Medical Center, Dallas, Texas, United States of America; Virginia Commonwealth University, UNITED STATES

## Abstract

It is well established that lung tumors induce the formation of lymphatic vessels. However, the molecular mechanisms controlling tumor lymphangiogenesis in lung cancer have not been fully delineated. In the present study, we identify a panel of non-small cell lung cancer (NSCLC) cell lines that induce lymphangiogenesis and use genome-wide mRNA expression to characterize the molecular mechanisms regulating tumor lymphangiogenesis. We show that Calu-1, H1993, HCC461, HCC827, and H2122 NSCLC cell lines form tumors that induce lymphangiogenesis whereas Calu-3, H1155, H1975, and H2073 NSCLC cell lines form tumors that do not induce lymphangiogenesis. By analyzing genome-wide mRNA expression data, we identify a 17-gene expression signature that distinguishes lymphangiogenic from non-lymphangiogenic NSCLC cell lines. Importantly, VEGF-C is the only lymphatic growth factor in this expression signature and is approximately 50-fold higher in the lymphangiogenic group than in the non-lymphangiogenic group. We show that forced expression of VEGF-C by H1975 cells induces lymphangiogenesis and that knockdown of VEGF-C in H1993 cells inhibits lymphangiogenesis. Additionally, we demonstrate that the triple angiokinase inhibitor, nintedanib (small molecule that blocks all FGFRs, PDGFRs, and VEGFRs), suppresses tumor lymphangiogenesis in H1993 tumors. Together, these data suggest that VEGF-C is the dominant driver of tumor lymphangiogenesis in NSCLC and reveal a specific therapy that could potentially block tumor lymphangiogenesis in NSCLC patients.

## Introduction

Lung cancer is the leading cause of cancer death among men and women in the United States [1]. Lung cancer patients typically die from the effect of metastases on distant organs. Lung cancer cells usually appear in regional lymph nodes before they are observed in distant organs. For this reason, lymph nodes are thought to function as “canaries in a coal mine” and are evaluated in order to determine whether cancer cells have spread from their primary site [2]. The presence of cancer cells in lymph nodes is associated with a poor prognosis and is one of the most important predictors of patient outcome for non-small cell lung cancer (NSCLC) and other carcinomas [2, 3]. This clinical observation fueled intense research efforts to identify processes that control the lymphogenous spread of cancer and, in 2001, it was reported that lymphangiogenesis, which is the sprouting of new lymphatic vessels from pre-existing vessels, facilitates metastasis to lymph nodes [4–6]. This landmark finding ignited great interest in delineating the molecular mechanisms controlling tumor lymphangiogenesis.

Over the past 15 years, substantial progress has been made in the field of tumor lymphangiogenesis research. Growth factors such as Adrenomedullin, Angiopoietin-1, Angiopoietin-2, HGF, Netrin-4, PDGF-BB, VEGF-A, VEGF-C, and VEGF-D have all been reported to promote tumor lymphangiogenesis [4–12]. Despite this progress, the precise mechanisms governing tumor lymphangiogenesis remain incompletely understood. This is in part because many studies on tumor lymphangiogenesis use cell lines that have been genetically engineered to overexpress a lymphatic growth factor [4–12]. Although the evaluation of genetically modified cell lines has provided valuable information on the role lymphatic vessels serve in tumors, they have not shed light on the precise mechanisms by which cancer cells induce the formation of lymphatic vessels. A better understanding of the molecular mechanisms controlling tumor lymphangiogenesis is needed in order to develop therapies that could potentially prevent the dissemination of cancer and improve the clinical outcome of patients with early stage disease. Therefore, we set out to identify a panel of cell lines that induce lymphangiogenesis and to use genome-wide mRNA expression data to identify the molecular mechanisms governing tumor lymphangiogenesis in NSCLC.

## Results

### Identification of lymphangiogenic and non-lymphangiogenic NSCLC cell lines

**Fig 1:**
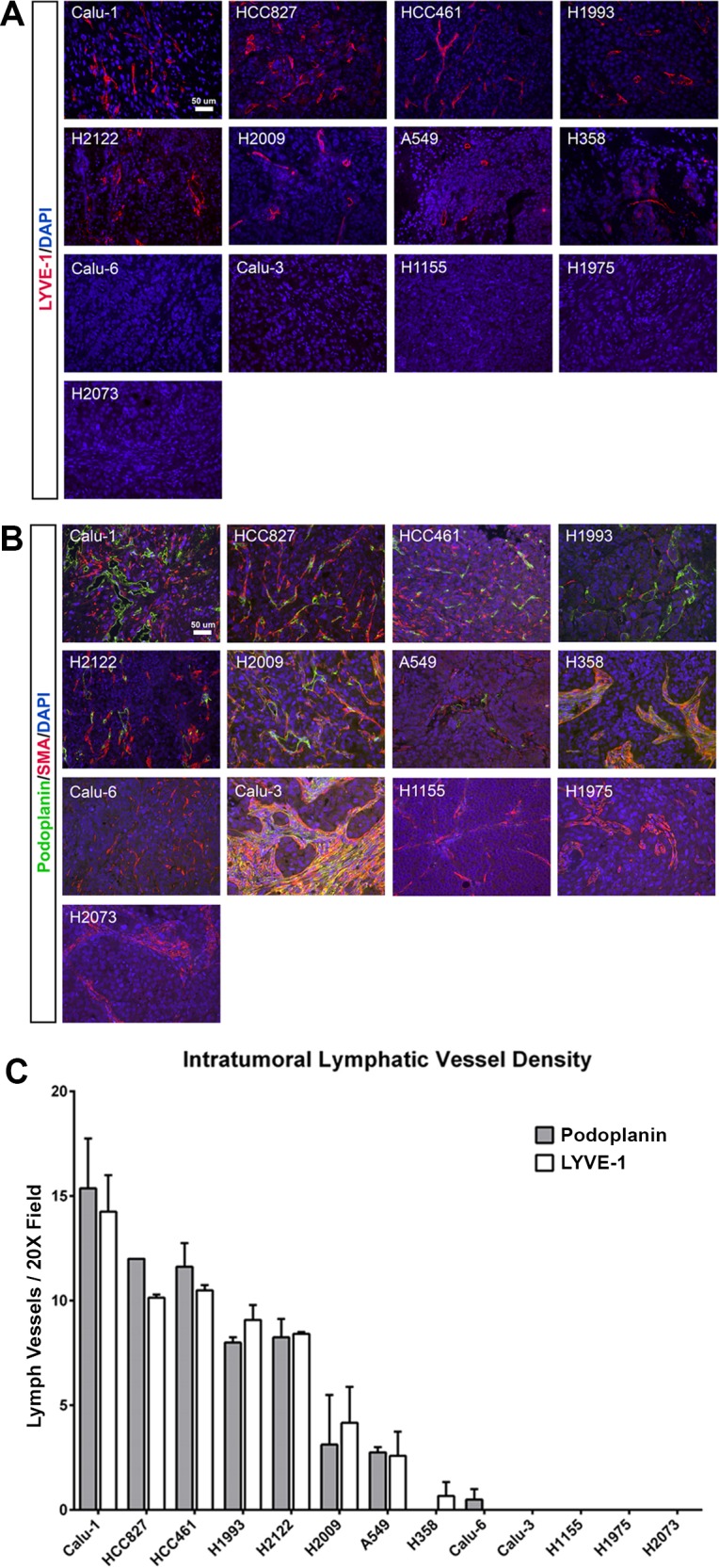
Identification of lymphangiogenic and non-lymphangiogenic NSCLC cell lines. **(A)** Representative images of lung tumor xenografts stained with an antibody against LYVE-1 (red) **(B)** Representative images of lung tumor xenografts stained with antibodies against podoplanin (green) and smooth muscle actin (SMA, red). **(C)** Graph showing intratumoral lymphatic vessel density for podoplanin and LYVE-1 positive lymphatic vessels in NSCLC xenografts grown in mice. We classified Calu-1, HCC827, HCC461, H1993, and H2122 cells as being lymphangiogenic and Calu-3, H1155, H1975, and H2073 as being non-lymphangiogenic. Graph shows mean ± SEM.

To identify NSCLC cell lines that induce lymphangiogenesis, we stained a panel of 13 NSCLC tumor xenograft samples from previous animal experiments with antibodies against LYVE-1 and podoplanin (**Fig 1**). These are two commonly assessed markers of lymphatic endothelial cells. An antibody against smooth muscle actin (SMA) was included in the podoplanin stain to help distinguish podoplanin-positive lymphatic vessels (podoplanin+;SMA-) from podoplanin-positive fibroblasts (podoplanin+;SMA+). The extent of lymphangiogenesis was quantified by counting the number of intratumoral lymphatic vessels per microscopic field and tumors were classified as being lymphangiogenic if they contained more than 5 lymphatic vessels per microscopic field or non-lymphangiogenic if they completely lacked intratumoral lymphatic vessels. Through this analysis, we were able to identify a panel of lymphangiogenic (Calu-1, H1993, HCC461, HCC827, and H2122) and non-lymphangiogenic NSCLC cell lines (Calu-3, H1155, H1975, and H2073) (**Fig 1**).

### VEGF-C regulates lymphangiogenesis by NSCLC cells

**Fig 2:**
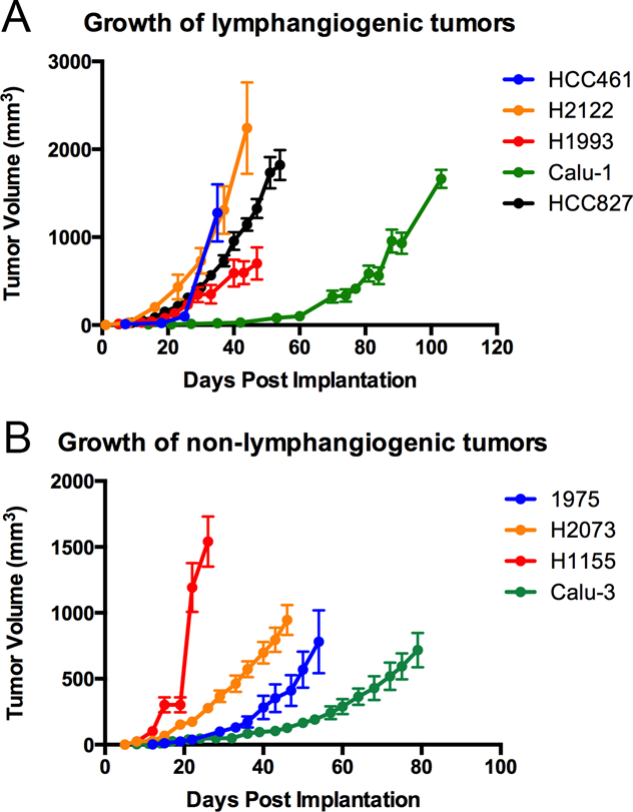
Growth curves for lymphangiogenic and non-lymphangiogenic tumors. **(A)** Graph showing the growth of lymphangiogenic (Calu-1, HCC827, HCC461, H1993, and H2122) tumors. **(B)** Graph showing the growth of non-lymphangiogenic (Calu-3, H1155, H1975, and H2073) tumors. Graph shows mean ± SEM.

After identifying lymphangiogenic and non-lymphangiogenic NSCLC cell lines, we set out to find differences between these two groups. We found that there was no obvious difference in the growth rate of lymphangiogenic and non-lymphangiogenic subcutaneous xenografts (**Fig 2**). We also found that the ability of a cell line to induce lymphangiogenesis was not related to its subtype classification (adenocarcinoma, squamous cell carcinoma, or large cell), site of origin (primary tumor versus metastasis), or mutation status (**Tables 1 and 2**).

**Table 1:** Characteristics of lymphangiogenic and non-lymphangiogenic NSCLC cell lines.

Cell Line	Tumor Type	Tumor Subtype	Tumor Source	Anatomical Site
**Calu-1**	NSCLC	Squamous Cell Carcinoma	metastasis	pleura
**HCC461**	NSCLC	Adenocarcinoma	primary	left upper lung
**HCC827**	NSCLC	Adenocarcinoma	primary	right lung
**H1993**	NSCLC	Adenocarcinoma	metastasis	lymph node
**H2122**	NSCLC	Adenocarcinoma	metastasis	pleural effusion
**Calu-3**	NSCLC	Adenocarcinoma	metastasis	pleural effusion
**H1155**	NSCLC	Large Cell	metastasis	lymph node
**H1975**	NSCLC	Adenocarcinoma	primary	lung
**H2073**	NSCLC	Adenocarcinoma	primary	lung

**Table 2:** Mutation status of lymphangiogenic and non-lymphangiogenic NSCLC cell lines.

	Lymphangiogenic					Non-Lymphangiogenic			
Gene	Calu-1	HCC827	HCC461	H1993	H2122	Calu-3	H1155	H1975	H2073
**TP53**	MUT	MUT	UNKNOWN	MUT	MUT	MUT	MUT	MUT	MUT
**CDKN2A**	WT	WT	UNKNOWN	WT	MUT	MUT	WT	MUT	WT
**KRAS**	MUT	WT	MUT	WT	MUT	WT	MUT	WT	WT
**PTEN**	WT	WT	UNKNOWN	WT	WT	WT	MUT	WT	WT
**RB1**	WT	WT	UNKNOWN	WT	WT	WT	MUT	WT	MUT
**BRAF**	WT	WT	WT	WT	WT	WT	WT	WT	WT
**PIK3CA**	WT	WT	WT	WT	WT	WT	WT	MUT	WT
**NRAS**	WT	WT	UNKNOWN	WT	WT	WT	WT	WT	WT
**STK11**	WT	WT	UNKNOWN	MUT	MUT	WT	WT	WT	MUT
**EGFR**	WT	MUT	WT	WT	WT	WT	WT	MUT	WT

**Fig 3:**
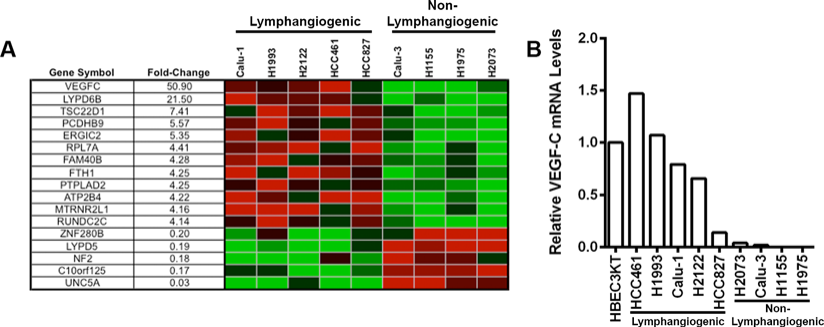
VEGF-C is differentially expressed between lymphangiogenic and non-lymphangiogenic NSCLC cell lines. **(A)** Microarray results showing genes differentially expressed between lymphangiogenic (Calu-1, HCC827, HCC461, H1993, and H2122) and non-lymphangiogenic (Calu-3, H1155, H1975, and H2073) cells. **(B)** Quantitative PCR results showing that VEGF-C is expressed at a higher level in lymphangiogenic than in non-lymphangiogenic NSCLC cell lines. VEGF-C values are normalized to the housekeeping gene GAPDH. Values for the NSCLC cell lines are normalized to an immortalized human bronchial epithelial cell line (HBEC3KT).

We then analyzed genome-wide mRNA expression data to identify genes differentially expressed between lymphangiogenic (Calu-1, H1993, HCC461, HCC827, and H2122) and non-lymphangiogenic (Calu-3, H1155, H1975, and H2073) cells. This analysis generated a 17-gene expression signature that distinguished lymphangiogenic from non-lymphangiogenic NSCLC cells (**Fig 3**). Vascular endothelial growth factor C (VEGF-C), a ligand of the receptor tyrosine kinases VEGFR2 and VEGFR3, was the only gene in this signature reported to stimulate lymphangiogenesis and was approximately 50-fold higher in the lymphangiogenic group than the non-lymphangiogenic group. We confirmed that VEGF-C was expressed at a higher level in lymphangiogenic cells than non-lymphangiogenic cells by quantitative PCR (**Fig 3**).

**Fig 4:**
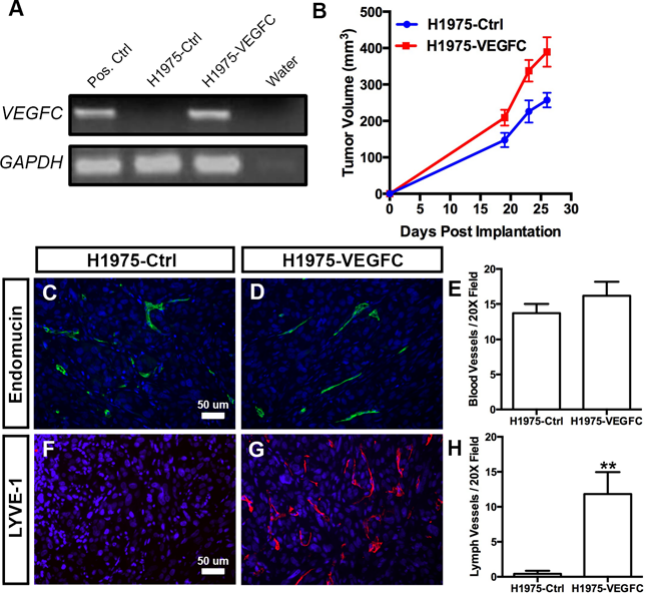
Forced expression of VEGF-C promotes tumor lymphangiogenesis. **(A)** RT-PCR results showing that H1975-VEGFC cells, but not H1975-Ctrl cells, express VEGF-C. **(B)** Tumor growth curves for mice injected subcutaneously with either H1975-Ctrl or H1975-VEGFC cells. **(C,D)** Representative images of H1975-Ctrl and H1975-VEGFC tumor sections stained with an anti-endomucin antibody. **(E)** The number of blood vessels per microscopic field is not significantly different between H1975-Ctrl tumors (13.75 ± 1.263, n = 7) and H1975-VEGFC tumors (16.18 ± 1.998, n = 7). **(F,G)** Representative images of H1975-Ctrl and H1975-VEGFC tumor sections stained with an anti-LYVE-1 antibody. **(H)** There are significantly more lymphatic vessels per microscopic field in H1975-VEGFC tumors (11.83 ± 3.125, n = 7) than in H1975-Ctrl tumors (0.4286 ± 0.4286 N = 7). ** *P* < 0.01.

To determine whether VEGF-C expression was sufficient to induce lymphangiogenesis by NSCLC cells, we genetically engineered H1975 cells to stably express either red fluorescent protein (RFP; H1975-Ctrl) or full length human VEGF-C (H1975-VEGFC). Reverse-transcription PCR analysis showed that H1975-VEGFC cells expressed a high level of VEGF-C (**Fig 4**). Additionally, quantitative PCR analysis showed that the level of VEGF-C mRNA is approximately 3-fold higher in H1975-VEGFC cells than H1993 cells (data not shown). We injected these cells into the flanks of NOD/SCID mice and found that H1975-VEGFC tumors grew slightly faster than H1975-Ctrl tumors (**Fig 4**). The density of intratumoral blood vessels was not significantly different between H1975-VEGFC tumors (16.18 ± 1.998, n = 7) and H1975-Ctrl tumors (13.75 ± 1.263, n = 7; **Fig 4**). However, the density of intratumoral lymphatic vessels was significantly greater in H1975-VEGFC tumors (11.83 ± 3.125, n = 7) than H1975-Ctrl tumors (0.4286 ± 0.4286 N = 7; **Fig 4**). These data show that forced expression of VEGF-C is sufficient to induce lymphangiogenesis by a NSCLC cell line.

**Fig 5:**
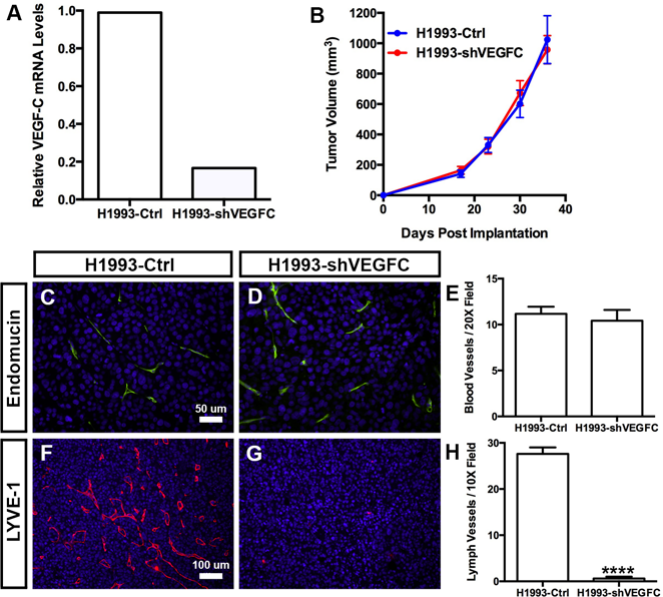
Knockdown of VEGF-C inhibits tumor lymphangiogenesis. **(A)** qPCR results showing that H1993-shVEGFC cells express a lower level of VEGF-C than H1993-Ctrl cells. **(B)** Tumor growth curves for mice injected subcutaneously with either H1993-Ctrl or H1993-shVEGFC cells. **(C,D)** Representative images of H1993-Ctrl and H1993-shVEGFC tumor sections stained with an anti-endomucin antibody. **(E)** The number of blood vessels per microscopic field is not significantly different between H1993-Ctrl tumors (11.17 ± 0.7817, n = 6) and H1993-shVEGFC tumors (10.42 ± 1.182, n = 7). **(F,G)** Representative images of H1993-Ctrl and H1993-shVEGFC tumor sections stained with an anti-LYVE-1 antibody. **(H)** The density of intratumoral lymphatic vessels is significantly lower in H1993-shVEGFC tumors (0.61 ± 0.400, n = 7) than H1993-Ctrl tumors (27.61 ± 1.391, n = 6). **** *P* < 0.0001.

To determine whether VEGF-C expression was required for NSCLC cells to induce lymphangiogenesis, we genetically engineered H1993 cells to stably express either green fluorescent protein (GFP; H1993-Ctrl) or an shRNA against VEGF-C (H1993-shVEGFC). Efficient knockdown of VEGF-C in H1993-shVEGFC cells was shown by quantitative PCR (**Fig 5**). We found that there was no difference in growth between H1993-shVEGFC and H1993-Ctrl tumors (**Fig 5**) and that the density of intratumoral blood vessels was not significantly different between H1993-shVEGFC tumors (10.42 ± 1.182, n = 7) and H1993-Ctrl tumors (11.17 ± 0.7817, n = 6; **Fig 5**). However, the density of intratumoral lymphatic vessels was significantly lower in H1993-shVEGFC tumors (0.61 ± 0.400, n = 7) than H1993-Ctrl tumors (27.61 ± 1.391, n = 6; **Fig 5**). These results show that VEGF-C is required for NSCLC cells to induce tumor lymphangiogenesis.

### Nintedanib inhibits tumor lymphangiogenesis

**Fig 6:**
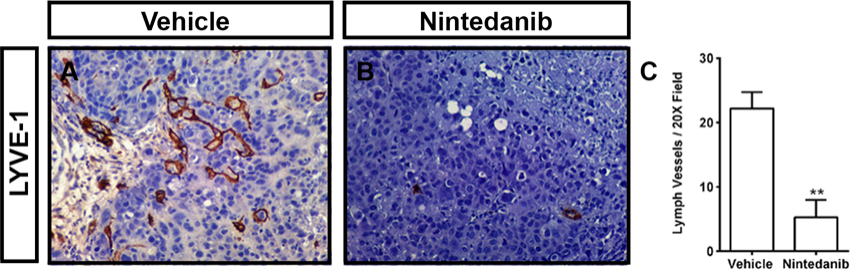
Nintedanib inhibits tumor lymphangiogenesis. **(A,B)** Representative images of LYVE-1-stained sections of H1993 tumors from vehicle and nintedanib treated mice. **(C)** The density of lymphatic vessels in H1993 xenografts is significantly lower in nintedanib treated mice (5.254 ± 2.745, n = 5) than in vehicle treated mice (22.19 ± 2.536, n = 6). ** *P* < 0.01.

Next, we sought to determine whether inhibition of the VEGF-C/VEGFR3 signaling axis with a clinically relevant compound could suppress tumor lymphangiogenesis. Nintedanib is a small molecule inhibitor that blocks all FGFRs (IC_50_ = 37–108 nM), PDGFRs (IC_50_ = 59–65 nM), and VEGFRs (IC_50_ = 13–34 nM) by binding to the ATP-binding site in the kinase domain of the receptors [13]. Nintedanib has previously been shown to block tumor angiogenesis and growth in several mouse models [13, 14]. Additionally, combination therapy of nintedanib with docetaxel has been reported to prolong the survival of stage III/IV NSCLC patients previously treated with a platinum-based therapy [15]. To determine whether nintedanib could block tumor lymphangiogenesis, we analyzed tumors from a previous study that evaluated the effect of nintedanib on the growth of H1993 tumors [14]. We found that the density of intratumoral lymphatic vessels was significantly lower in nintedanib treated H1993 tumors (5.254 ± 2.745, n = 5) than vehicle treated H1993 tumors (22.19 ± 2.536, n = 6; **Fig 6**). These data show that nintedanib is effective at inhibiting tumor lymphangiogenesis.

### VEGF-C copy number variation influences VEGF-C expression

**Fig 7:**
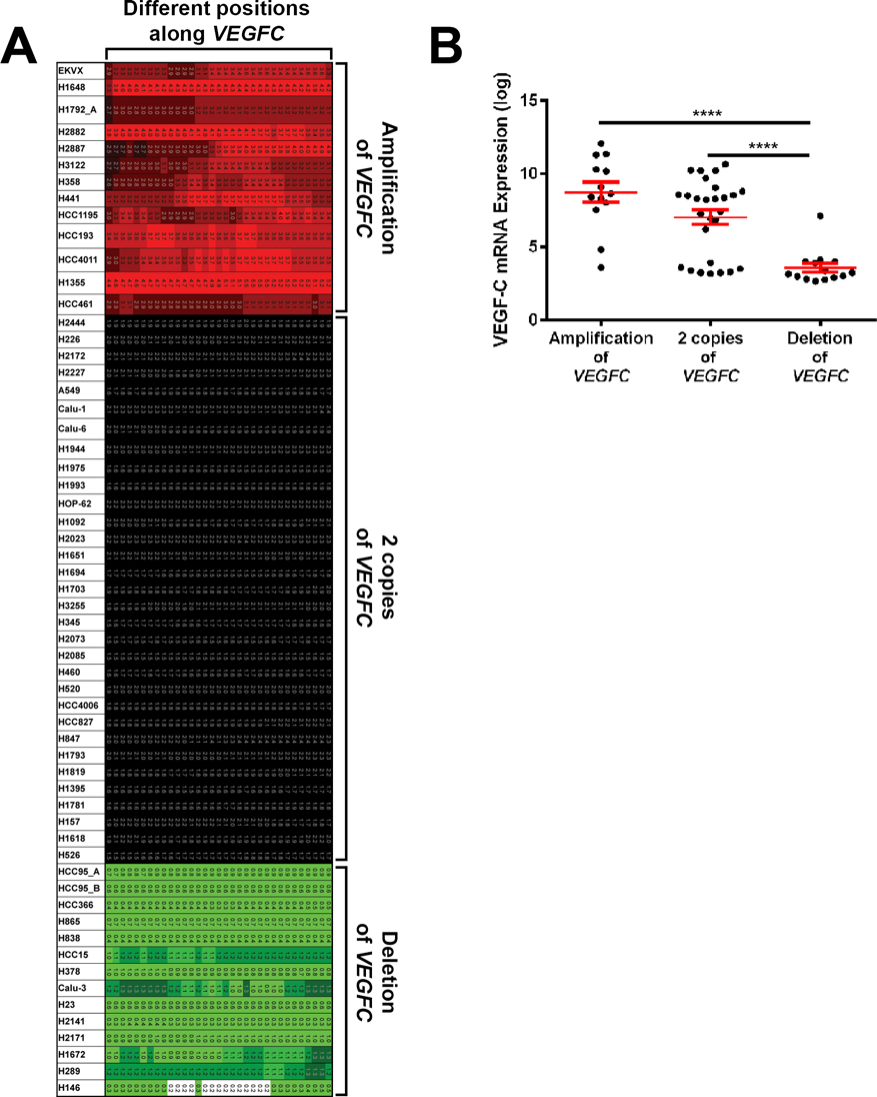
*VEGF-C copy number variation influences VEGF-C expression*. **(A)** Image showing *VEGFC* copy number variation for different lung cancer cell lines. Rows show data for individual lung cancer cell lines. Columns mark different positions along the *VEGFC* gene. Amplified positions are colored red, diploid positions are colored black, and deleted regions are colored green or white. **(B)** Graph showing log transformed *VEGF-C* mRNA levels from microarray data for cell lines that have more than 2 copies (range is between 3–5 copies of *VEGFC*), 2 copies, or less than 2 copies of the *VEGFC* gene. Graph shows mean ± SEM. **** *P* < 0.0001

Cancer cells frequently undergo genomic alterations that result in the amplification and deletion of genes. These genomic alterations can impact the expression of genes. To determine whether the *VEGF-C* gene is amplified or deleted in lung cancer cells, we analyzed SNP array data available for 59 lung cancer cell lines. This revealed that the *VEGF-C* gene was amplified in 22% (13/59; range between 3–5 copies of *VEGFC*), present as 2 copies in 54% (32/59), and deleted in 24% (14/59) of the lung cancer cell lines that we analyzed (**Fig 7**). To determine whether changes in the number of copies of the *VEGF-C* gene influenced the expression of *VEGF-C*, we evaluated log transformed microarray values for *VEGF-C* in this panel of 59 lung cancer cell lines. This revealed that the level of *VEGF-C* was significantly lower in cells that had deletion (3.582 ± 0.3033) of the *VEGF-C* gene compared to cells that had either 2 copies (7.029 ± 0.5023) or amplification (8.742 ± 0.6856) of the *VEGF-C* gene (**Fig 7**). These data show that *VEGF-C* copy number variation can affect the expression level of *VEGF-C*.

## Discussion

The study of molecularly annotated lung cancer cell lines has increased our understanding of the pathways driving tumorigenesis and has led to the identification of novel biomarkers and therapeutic targets for lung cancer. In the present study, we use a panel of molecularly annotated NSCLC cell lines to investigate the molecular mechanisms controlling tumor lymphangiogenesis. We show that VEGF-C expression regulates tumor lymphangiogenesis by NSCLC cells and that inhibition of VEGF-C-induced signaling with nintedandinb can block tumor lymphangiogenesis by NSCLC cells.

VEGF-C has emerged as a central figure in the field of lymphangiogenesis research. VEGF-C has been shown to be sufficient to induce tumor lymphangiogenesis by melanoma [16, 17], breast cancer [4, 12, 18], fibrosarcoma [17], and gastric carcinoma cells [19]. Additionally, inhibition of VEGF-C has been reported to suppress lymphangiogenesis by prostate [20, 21], pancreatic [22], breast [23–25], gastric [26], and lung cancer cells [27]. VEGF-C expression has also been reported to correlate with lymphatic vessel density in many different human tumors, including NSCLC [28–31]. We show that VEGF-C expression distinguishes NSCLC cell lines that induce lymphangiogenesis from NSCLC cell lines that do not induce lymphangiogenesis. Additionally, we show by overexpression and knockdown experiments that VEGF-C regulates tumor lymphangiogenesis by NSCLC cells. These findings further demonstrate the importance of VEGF-C in promoting tumor lymphangiogenesis and suggest that it is the dominant driver of tumor lymphangiogenesis in NSCLC.

Nintedanib is a small molecule tyrosine kinase inhibitor that blocks all FGF, PGDF, and VEGF receptors. Nintedanib was previously shown to display anti-cancer effects in a number of preclinical models of NSCLC and in NSCLC patients [14, 15]. We show that nintedanib can block tumor lymphangiogenesis in a mouse model of NSCLC. Although we show that nintedanib has anti-lymphangiogenic activity, this compound was previously reported to not inhibit tumor lymphangiogenesis in a transgenic mouse model of pancreatic neuroendocrine tumor (PNET) that was genetically engineered to overexpress VEGF-C [32]. The lack of an anti-lymphangiogenic effect by nintedanib in *Rip1-Tag2;Rip1-Vegfc* transgenic mice could be because an extensive network of irregular lymphatic vessels might have been present in the pancreas prior to the start of therapy. It has been reported that newly formed lymphatic vessels can persist for a long period of time after the withdrawal of VEGF-C or in the face of anti-lymphangiogenic therapy [33]. Therefore, any lymphatic vessels that formed in *Rip1-Tag2;Rip1-Vegfc* transgenic mice prior to the start of therapy could potentially be resistant to the anti-lymphangiogenic effects of nintedanib. Alternatively, the difference between our findings and those of Bill et al., (2015) could be because we examined different tumor types or because we used an unmanipulated cell line and they used a genetically engineered model to overexpress VEGF-C.

The molecular mechanisms controlling *VEGF-C* mRNA levels are not well understood. We show that changes in the number of copies of the *VEGF-C* gene affect the expression of *VEGF-C*. We found that lung cancer cell lines that have lost one of their copies of the *VEGF-C* gene tend to express a low level of *VEGF-C*. However, additional mechanisms also likely control the expression of VEGF-C by NSCLC cells. The MAPK, mTOR, and NF-kB signaling pathways have all been reported to control VEGF-C expression by cancer cells [34–37]. These pathways may also play a part in controlling the expression of VEGF-C by NSCLC cells. Future studies with our panel of cell lines will help determine the potential role of these and other pathways in controlling the expression of VEGF-C by NSCLC cells.

In conclusion, the results of this study demonstrate that VEGF-C is a critical regulator of tumor lymphangiogenesis in NSCLC and show that nintedanib inhibits tumor lymphangiogenesis. These findings shed light on the molecular mechanisms driving tumor lymphangiogenesis and have the potential to influence the design of future clinical trials aimed at blocking the spread of early stage NSCLC.

## Materials and Methods

### Ethics Statement

The animal experiments described in this manuscript were carried out in accordance with an animal protocol (APN 2013–0121) approved by the Institutional Animal Care and Use Committee (IACUC) of UT Southwestern Medical Center. All mice in this study were purchased from an on-campus supplier. Mice were maintained in ventilated microisolater cages in a pathogen-free facility and were fed a standard irradiated diet ad libitum. Mice were provided nestlets and igloos as enrichment items. Mice were monitored for signs of distress such as lethargy and changes in fur appearance. If mice appeared severely ill or moribund, they would be euthanized by an overdose of carbon dioxide followed by cervical dislocation. No mice became severely ill or died prior to the experimental endpoint. The method of euthanasia for the experimental endpoints consisted of an inhalant overdose of carbon dioxide or isoflurane followed by cervical dislocation. These methods are consistent with the recommendations of the American Veterinary Medical Association (AVMA) Guidelines on Euthanasia.

### Cell lines

The human bronchial epithelial cell line (HBEC3KT) and most of the human NSCLC cell lines used in this study (H1993, HCC461, HCC827, H2122, H1155, H1975, and H2073) were established in the laboratories of Dr. Adi Gazdar and Dr. John Minna [38–40]. The NSCLC cell lines Calu-1 and Calu-3 were purchased from the American Type Culture Collection (ATCC, Manassas, VA). The NSCLC cell lines were cultured in DMEM + 10% FBS under standard conditions (5% CO_2_ at 37°C). The HBEC3KT cell line was cultured in keratinocyte serum-free media containing 5 ng/mL of EGF and 50 μg/mL of bovine pituitary extract under standard conditions (5% CO_2_ at 37°C). All NSCLC cell lines were DNA-fingerprinted and mycoplasma-tested.

### Quantitative PCR and RT-PCR

RNA was isolated from the various cell lines with an RNeasy mini kit (Qiagen, cat no: 74104) and cDNA was generated with an iScript cDNA synthesis kit (BioRad, cat no: 170–8890). Gene-specific TaqMan probes were used to analyze the levels of *VEGFC* (Applied Biosystems, Hs01099206_m1) and *GAPDH* (Applied Biosystems, Hs02758991_g1) and the comparative *C*_t_ method was used to calculate relative mRNA expression levels. The following primers were used in RT-PCR reactions to amplify *VEGF-C* (5’- GTTCGTACATGGCCGTCTGT-3’ and 5’- GGACCAAACAAGGAGCTGGA-3’) and *GAPDH* (5’- CTCTGCTCCTCCTGTTCGAC-3’ and 5’- GTTAAAAGCAGCCCTGGTGA-3’).

### Generation of stable cell lines

To overexpress VEGF-C in a non-lymphangiogenic NSCLC cell line, we infected H1975 cells with commercially available lentiviral particles that contain a plasmid that expresses full length human VEGF-C (Precision LentiORF VEGFC w/ Stop Codon; Open Biosystems, cat no: OHS5899-202618255). To generate control cells, we infected H1975 cells with lentiviral particles that express RFP (Precision LentiORF RFP Positive Control; Open Biosystems, cat no: OHS5833). Cells were grown in media containing blasticidin (30 ug/ml) for several weeks to select for stably transfected cells.

To stably knockdown VEGF-C in a lymphangiogenic NSCLC cell line, we infected H1993 cells with commercially available lentiviral particles that express an shRNA targeting VEGF-C (TRCN0000425238; Sigma, cat no: SHCLNV-NM_005429). To generate control cells, we infected H1993 cells with lentiviral particles that express GFP (MISSION® TRC2 pLKO.5-puro-CMV-TurboGFP™ Positive Control Transduction Particles; Sigma, cat no: SHC203V). Cells were grown in media containing puromycin (1 ug/ml) for several weeks to select for stably transfected cells.

### Animal experiments

NOD/SCID mice received a subcutaneous injection of 1 million H1975-Ctrl, H1975-VEGFC, H1993-Ctrl, or H1993-shVEGFC cells. Mice were weighed and tumors were measured twice a week. Tumor volumes were calculated with the formula *V = (a*^*2*^ *x b)/2*, with *a* and *b* representing the small and large tumor diameters, respectively. Mice were euthanized before their tumors reached 1,500 mm^3^ and their tissues were collected for histological analysis.

### Antibodies

The following primary antibodies were used for immunohistochemistry or immunofluorescence staining of tumors: goat anti-LYVE-1 (R&D Systems, cat no. AF2125), rat anti-endomucin (Santa Cruz, cat no. sc-65495), Cy3-conjugated mouse anti-smooth muscle actin (Sigma, cat no. C6198), and hamster anti-podoplanin (abcam, cat no. ab11936). All secondary antibodies were purchased from Jackson ImmunoResearch.

### Immunofluorescence/immunohistochemistry staining

Slides were de-paraffinized with xylene and rehydrated through a descending EtOH series. Antigen retrieval was performed with 0.01 M citric acid (pH 6.0) in a pressure cooker. Slides were then washed with PBS and blocked for 1 hour with TBST + 20% Aquablock. Primary antibodies diluted in TBST + 5% BSA were then added and allowed to incubate overnight at 4°C. Slides were washed with TBST then secondary antibodies diluted in TBST + 5% BSA were added and allowed to incubate for 1 hour at room temperature. Slides were then washed again with TBST and coverslips were mounted with ProLong Gold plus DAPI. Immunohistochemistry was performed using a similar protocol except endogenous peroxidase activity was blocked by incubating slides with hydrogen peroxide diluted in MeOH and signal was detected via the DAB chromogen system (Dako, cat no. K3468).

### Quantitative analysis of blood and lymphatic vessels

Slides were analyzed with a Nikon Eclipse E600 microscope and images were captured using NIS-Elements imaging software. To analyze blood vessels, 4 pictures were taken of each tumor and the number of vessels was counted per microscopic field. To analyze lymphatic vessels, 3–5 pictures were taken of “hot spots” in each tumor and the number of vessels was counted per microscopic field.

### Mutation status of cell lines

The mutation status of the cell lines was determined by analyzing data from published sources [38–42] and COSMIC (Sanger Institute, UK).

### Microarray analysis

RNA quality and concentration were checked by the Bio-Rad Experion Bioanalyzer per manufacturer's protocol. 500 ng of total RNA from each sample was used to label the cRNA probes by Ambion Illumina TotalPrep RNA Amplification kit (cat no: IL1791). 1.5 ug of the amplified and labeled cRNA probes was hybridized to Illumina Human WG-6 v3.0 Expression BeadChip (cat no: BD-101-0203) overnight at 58°C, then washed, blocked and detected by streptavidin-Cy3 per manufacturer's protocol. After drying, the chips were scanned by Illumina iScan system. Bead-level data were obtained, and pre-processed using the R package mbcb for background correction and probe summarization (Ding et al, Nucl Acids Res, 36:e58, 2008). Pre-processed data were then quantile-normalized and log-transformed. Microarray results for the NSCLC cell lines were previously published [43] and archived at the Gene Expression Omnibus repository (http://www.ncbi.nlm.nih.gov/geo/; GEO accession number: GSE32036).

Differentially expressed genes between two classes of samples were determined by calculating fold change and T-test *P* values and using arbitrary cutoffs for selection (e.g. > 4 fold change and *P* < 0.01). Because of the low number of samples in each class, we did not adjust the *P* values with multiple testing correction.

### SNP arrays

Whole genome single nucleotide polymorphism (SNP) array profiling was done with the Illumina Human1M-Duo DNA Analysis BeadChip (Illumina, Inc.)[44]. Processing was done with Illumina BeadStudio and DNA copy number was derived from the “Log R Ratio”, which measures the relative probe intensity compared with normal diploid controls

### Statistical analysis

Data were analyzed using GraphPad Prism statistical analysis software (Version 6.0). All results are expressed as mean ± SEM. For experiments with two groups, unpaired student’s T-tests were performed to test means for significance. For experiments with more than two groups, differences were assessed by ANOVA followed by Tukey’s multiple comparisons test. Data were considered significant at *P* < 0.05.

## References

[1] SEER Cancer Statistics Factsheets: Lung and Bronchus Cancer. National Cancer Institute Bethesda M, http://seer.cancer.gov/statfacts/html/lungb.html.

[2] Mountain CF, Dresler CM. Regional lymph node classification for lung cancer staging. Chest. 1997;111(6):1718–23. . 9187199 10.1378/chest.111.6.1718

[3] Oda M, Watanabe Y, Shimizu J, Murakami S, Ohta Y, Sekido N, et al Extent of mediastinal node metastasis in clinical stage I non-small-cell lung cancer: the role of systematic nodal dissection. Lung cancer. 1998;22(1):23–30. . 9869104 10.1016/s0169-5002(98)00070-1

[4] Skobe M, Hawighorst T, Jackson DG, Prevo R, Janes L, Velasco P, et al Induction of tumor lymphangiogenesis by VEGF-C promotes breast cancer metastasis. Nature medicine. 2001;7(2):192–8. Epub 2001/02/15. 10.1038/84643 . 11175850

[5] Mandriota SJ, Jussila L, Jeltsch M, Compagni A, Baetens D, Prevo R, et al Vascular endothelial growth factor-C-mediated lymphangiogenesis promotes tumour metastasis. The EMBO journal. 2001;20(4):672–82. 10.1093/emboj/20.4.672 11179212 PMC145430

[6] Stacker SA, Caesar C, Baldwin ME, Thornton GE, Williams RA, Prevo R, et al VEGF-D promotes the metastatic spread of tumor cells via the lymphatics. Nature medicine. 2001;7(2):186–91. 10.1038/84635 . 11175849

[7] Karpinich NO, Kechele DO, Espenschied ST, Willcockson HH, Fedoriw Y, Caron KM. Adrenomedullin gene dosage correlates with tumor and lymph node lymphangiogenesis. FASEB journal: official publication of the Federation of American Societies for Experimental Biology. 2013;27(2):590–600. 10.1096/fj.12-214080 23099649 PMC3545524

[8] Fagiani E, Lorentz P, Kopfstein L, Christofori G. Angiopoietin-1 and -2 exert antagonistic functions in tumor angiogenesis, yet both induce lymphangiogenesis. Cancer research. 2011;71(17):5717–27. 10.1158/0008-5472.CAN-10-4635 . 21778249

[9] Cao R, Bjorndahl MA, Gallego MI, Chen S, Religa P, Hansen AJ, et al Hepatocyte growth factor is a lymphangiogenic factor with an indirect mechanism of action. Blood. 2006;107(9):3531–6. 10.1182/blood-2005-06-2538 . 16424394

[10] Cao R, Bjorndahl MA, Religa P, Clasper S, Garvin S, Galter D, et al PDGF-BB induces intratumoral lymphangiogenesis and promotes lymphatic metastasis. Cancer cell. 2004;6(4):333–45. 10.1016/j.ccr.2004.08.034 . 15488757

[11] Larrieu-Lahargue F, Welm AL, Thomas KR, Li DY. Netrin-4 induces lymphangiogenesis in vivo. Blood. 2010;115(26):5418–26. 10.1182/blood-2009-11-252338 20407033 PMC2902137

[12] Karpanen T, Egeblad M, Karkkainen MJ, Kubo H, Yla-Herttuala S, Jaattela M, et al Vascular endothelial growth factor C promotes tumor lymphangiogenesis and intralymphatic tumor growth. Cancer research. 2001;61(5):1786–90. . 11280723

[13] Hilberg F, Roth GJ, Krssak M, Kautschitsch S, Sommergruber W, Tontsch-Grunt U, et al BIBF 1120: triple angiokinase inhibitor with sustained receptor blockade and good antitumor efficacy. Cancer research. 2008;68(12):4774–82. 10.1158/0008-5472.CAN-07-6307 . 18559524

[14] Kutluk Cenik B, Ostapoff KT, Gerber DE, Brekken RA. BIBF 1120 (nintedanib), a triple angiokinase inhibitor, induces hypoxia but not EMT and blocks progression of preclinical models of lung and pancreatic cancer. Molecular cancer therapeutics. 2013;12(6):992–1001. 10.1158/1535-7163.MCT-12-0995 23729403 PMC3681897

[15] Reck M, Kaiser R, Mellemgaard A, Douillard JY, Orlov S, Krzakowski M, et al Docetaxel plus nintedanib versus docetaxel plus placebo in patients with previously treated non-small-cell lung cancer (LUME-Lung 1): a phase 3, double-blind, randomised controlled trial. The Lancet Oncology. 2014;15(2):143–55. 10.1016/S1470-2045(13)70586-2 . 24411639

[16] Skobe M, Hamberg LM, Hawighorst T, Schirner M, Wolf GL, Alitalo K, et al Concurrent induction of lymphangiogenesis, angiogenesis, and macrophage recruitment by vascular endothelial growth factor-C in melanoma. Am J Pathol. 2001;159(3):893–903. Epub 2001/09/11. doi: S0002-9440(10)61765-8 [pii] 10.1016/S0002-9440(10)61765-8 11549582 PMC1850477

[17] Hoshida T, Isaka N, Hagendoorn J, di Tomaso E, Chen YL, Pytowski B, et al Imaging steps of lymphatic metastasis reveals that vascular endothelial growth factor-C increases metastasis by increasing delivery of cancer cells to lymph nodes: therapeutic implications. Cancer research. 2006;66(16):8065–75. 10.1158/0008-5472.CAN-06-1392 . 16912183

[18] Mattila MM, Ruohola JK, Karpanen T, Jackson DG, Alitalo K, Harkonen PL. VEGF-C induced lymphangiogenesis is associated with lymph node metastasis in orthotopic MCF-7 tumors. International journal of cancer Journal international du cancer. 2002;98(6):946–51. . 11948478 10.1002/ijc.10283

[19] Yanai Y, Furuhata T, Kimura Y, Yamaguchi K, Yasoshima T, Mitaka T, et al Vascular endothelial growth factor C promotes human gastric carcinoma lymph node metastasis in mice. Journal of experimental & clinical cancer research: CR. 2001;20(3):419–28. . 11718224

[20] Burton JB, Priceman SJ, Sung JL, Brakenhielm E, An DS, Pytowski B, et al Suppression of prostate cancer nodal and systemic metastasis by blockade of the lymphangiogenic axis. Cancer research. 2008;68(19):7828–37. 10.1158/0008-5472.CAN-08-1488 18829538 PMC2800077

[21] Wong SY, Haack H, Crowley D, Barry M, Bronson RT, Hynes RO. Tumor-secreted vascular endothelial growth factor-C is necessary for prostate cancer lymphangiogenesis, but lymphangiogenesis is unnecessary for lymph node metastasis. Cancer research. 2005;65(21):9789–98. 10.1158/0008-5472.CAN-05-0901 . 16267000

[22] Shi Y, Tong M, Wu Y, Yang Z, Hoffman RM, Zhang Y, et al VEGF-C ShRNA inhibits pancreatic cancer growth and lymphangiogenesis in an orthotopic fluorescent nude mouse model. Anticancer research. 2013;33(2):409–17. . 23393331

[23] Shibata MA, Shibata E, Morimoto J, Harada-Shiba M. Therapy with siRNA for Vegf-c but not for Vegf-d suppresses wide-spectrum organ metastasis in an immunocompetent xenograft model of metastatic mammary cancer. Anticancer research. 2013;33(10):4237–47. . 24122987

[24] Guo B, Zhang Y, Luo G, Li L, Zhang J. Lentivirus-mediated small interfering RNA targeting VEGF-C inhibited tumor lymphangiogenesis and growth in breast carcinoma. Anatomical record. 2009;292(5):633–9. 10.1002/ar.20893 . 19382240

[25] Chen Z, Varney ML, Backora MW, Cowan K, Solheim JC, Talmadge JE, et al Down-regulation of vascular endothelial cell growth factor-C expression using small interfering RNA vectors in mammary tumors inhibits tumor lymphangiogenesis and spontaneous metastasis and enhances survival. Cancer research. 2005;65(19):9004–11. 10.1158/0008-5472.CAN-05-0885 . 16204074

[26] Yao J, Da M, Guo T, Duan Y, Zhang Y. RNAi-mediated gene silencing of vascular endothelial growth factor-C inhibits tumor lymphangiogenesis and growth of gastric cancer in vivo in mice. Tumour biology: the journal of the International Society for Oncodevelopmental Biology and Medicine. 2013;34(3):1493–501. 10.1007/s13277-013-0674-6 . 23475632

[27] He Y, Kozaki K, Karpanen T, Koshikawa K, Yla-Herttuala S, Takahashi T, et al Suppression of tumor lymphangiogenesis and lymph node metastasis by blocking vascular endothelial growth factor receptor 3 signaling. Journal of the National Cancer Institute. 2002;94(11):819–25. . 12048269 10.1093/jnci/94.11.819

[28] Bo C, Xiaopeng D, Chuanliang P, Xiaogang Z. Expression of vascular endothelial growth factors C and D correlates with lymphangiogenesis and lymph node metastasis in lung adenocarcinoma. The Thoracic and cardiovascular surgeon. 2009;57(5):291–4. 10.1055/s-0029-1185625 . 19629892

[29] Feng Y, Wang W, Hu J, Ma J, Zhang Y, Zhang J. Expression of VEGF-C and VEGF-D as significant markers for assessment of lymphangiogenesis and lymph node metastasis in non-small cell lung cancer. Anatomical record. 2010;293(5):802–12. 10.1002/ar.21096 . 20225197

[30] Kadota K, Huang CL, Liu D, Ueno M, Kushida Y, Haba R, et al The clinical significance of lymphangiogenesis and angiogenesis in non-small cell lung cancer patients. European journal of cancer. 2008;44(7):1057–67. 10.1016/j.ejca.2008.03.012 . 18396396

[31] Sun JG, Wang Y, Chen ZT, Zhuo WL, Zhu B, Liao RX, et al Detection of lymphangiogenesis in non-small cell lung cancer and its prognostic value. Journal of experimental & clinical cancer research: CR. 2009;28:21 10.1186/1756-9966-28-21 19216806 PMC2647904

[32] Bill R, Fagiani E, Zumsteg A, Antoniadis H, Johansson D, Haefliger S, et al Nintedanib Is a Highly Effective Therapeutic for Neuroendocrine Carcinoma of the Pancreas (PNET) in the Rip1Tag2 Transgenic Mouse Model. Clinical cancer research: an official journal of the American Association for Cancer Research. 2015 10.1158/1078-0432.CCR-14-3036 . 26206868

[33] Yao LC, Testini C, Tvorogov D, Anisimov A, Vargas SO, Baluk P, et al Pulmonary lymphangiectasia resulting from vascular endothelial growth factor-C overexpression during a critical period. Circulation research. 2014;114(5):806–22. 10.1161/CIRCRESAHA.114.303119 24429550 PMC3969887

[34] Luangdilok S, Box C, Harrington K, Rhys-Evans P, Eccles S. MAPK and PI3K signalling differentially regulate angiogenic and lymphangiogenic cytokine secretion in squamous cell carcinoma of the head and neck. European journal of cancer. 2011;47(4):520–9. Epub 2010/11/16. doi: S0959-8049(10)00994-9 [pii] 10.1016/j.ejca.2010.10.009 . 21074412

[35] Kobayashi S, Kishimoto T, Kamata S, Otsuka M, Miyazaki M, Ishikura H. Rapamycin, a specific inhibitor of the mammalian target of rapamycin, suppresses lymphangiogenesis and lymphatic metastasis. Cancer science. 2007;98(5):726–33. Epub 2007/04/12. doi: CAS439 [pii] 10.1111/j.1349-7006.2007.00439.x . 17425689 PMC11158643

[36] Du Q, Jiang L, Wang X, Wang M, She F, Chen Y. Tumor necrosis factor-alpha promotes the lymphangiogenesis of gallbladder carcinoma through nuclear factor-kappaB-mediated upregulation of vascular endothelial growth factor-C. Cancer science. 2014;105(10):1261–71. 10.1111/cas.12504 25154789 PMC4462363

[37] Tsai PW, Shiah SG, Lin MT, Wu CW, Kuo ML. Up-regulation of vascular endothelial growth factor C in breast cancer cells by heregulin-beta 1. A critical role of p38/nuclear factor-kappa B signaling pathway. The Journal of biological chemistry. 2003;278(8):5750–9. 10.1074/jbc.M204863200 . 12471041

[38] Phelps RM, Johnson BE, Ihde DC, Gazdar AF, Carbone DP, McClintock PR, et al NCI-Navy Medical Oncology Branch cell line data base. Journal of cellular biochemistry Supplement. 1996;24:32–91. . 8806092 10.1002/jcb.240630505

[39] Shigematsu H, Lin L, Takahashi T, Nomura M, Suzuki M, Wistuba II, et al Clinical and biological features associated with epidermal growth factor receptor gene mutations in lung cancers. Journal of the National Cancer Institute. 2005;97(5):339–46. 10.1093/jnci/dji055 . 15741570

[40] Shigematsu H, Takahashi T, Nomura M, Majmudar K, Suzuki M, Lee H, et al Somatic mutations of the HER2 kinase domain in lung adenocarcinomas. Cancer research. 2005;65(5):1642–6. 10.1158/0008-5472.CAN-04-4235 . 15753357

[41] Yamamoto H, Shigematsu H, Nomura M, Lockwood WW, Sato M, Okumura N, et al PIK3CA mutations and copy number gains in human lung cancers. Cancer research. 2008;68(17):6913–21. 10.1158/0008-5472.CAN-07-5084 18757405 PMC2874836

[42] Modi S, Kubo A, Oie H, Coxon AB, Rehmatulla A, Kaye FJ. Protein expression of the RB-related gene family and SV40 large T antigen in mesothelioma and lung cancer. Oncogene. 2000;19(40):4632–9. 10.1038/sj.onc.1203815 . 11030152

[43] Byers LA, Diao L, Wang J, Saintigny P, Girard L, Peyton M, et al An epithelial-mesenchymal transition gene signature predicts resistance to EGFR and PI3K inhibitors and identifies Axl as a therapeutic target for overcoming EGFR inhibitor resistance. Clinical cancer research: an official journal of the American Association for Cancer Research. 2013;19(1):279–90. 10.1158/1078-0432.CCR-12-1558 23091115 PMC3567921

[44] Yang F, Tang X, Riquelme E, Behrens C, Nilsson MB, Giri U, et al Increased VEGFR-2 gene copy is associated with chemoresistance and shorter survival in patients with non-small-cell lung carcinoma who receive adjuvant chemotherapy. Cancer research. 2011;71(16):5512–21. 10.1158/0008-5472.CAN-10-2614 21724587 PMC3159530

